# Isolated Dissections of Superior Mesenteric and Celiac Arteries Associated With Aortic Ectasia

**DOI:** 10.7759/cureus.8657

**Published:** 2020-06-16

**Authors:** Ejaz Latif, Amr Fares, Zahoor Ahmed, Shameel Musthafa, Imran Mazhar

**Affiliations:** 1 General Surgery, Hamad Medical Corporation, Doha, QAT; 2 Vascular Surgery, Hamad Medical Corporation, Doha, QAT; 3 Radiology, Hamad Medical Corporation, Doha, QAT; 4 Acute Care Surgery, Hamad Medical Corporation, Doha, QAT; 5 General Surgery, Railway General Hospital, Rawalpindi, PAK

**Keywords:** arterial dissection, multiple visceral arterial dissection, celiac artery dissection, superior mesenteric artery dissection, computed tomography angiography

## Abstract

Arterial dissection is defined as a tear in the inner lining of arteries, leading to the passage of blood between the layers and resulting in a false lumen. Arterial dissection involving the aorta is commonly seen in clinical practice; however, dissections involving the celiac and superior mesenteric arteries are quite rare. Even rare are isolated multiple visceral arterial dissections.

A 59-year-old male with uncontrolled hypertension presented with epigastric pain. CT angiography revealed isolated dissection of the celiac and superior mesenteric arteries with ascending aortic ectasia, with no features of ischemia or organ dysfunction. He was managed conservatively with analgesics & anticoagulation. Repeat CT angiography after six months of anticoagulation therapy showed no progression of the disease.

Isolated multiple visceral arterial dissection is a rare vascular disease that requires a high index of suspicion to diagnose. CT angiography is a useful imaging modality that helps not only in diagnosis but also in choosing a treatment plan. Though treatment options are controversial, conservative treatment with anticoagulation should be considered in uncomplicated cases of visceral arterial dissections.

## Introduction

Arterial dissection (AD) is defined as a tear in the inner lining of arteries which allows passage of blood between the layers resulting in a false lumen. Isolated multiple ADs involving the celiac artery (CA) and the superior mesenteric artery (SMA) are quite rare and carry a significant risk of potentially lethal complications such as the progression of dissection, bowel ischemia, aneurysmal rupture, and massive bleeding [1-3]. A high index of suspicion is needed to accurately diagnose and treat this condition. There are various treatment modalities available, such as conservative management, endovascular procedures, and open surgical procedures. As there are no clear guidelines, optimal management is still debated. Herein, we describe conservative treatment in symptomatic isolated dissections of CA and SMA and discuss our findings and outcome.

## Case presentation

A 59-year-old male, smoker, and known case of hypertension presented to the emergency department with complaints of epigastric pain for two days’ duration. The pain started suddenly, initially mild, but later became severe, non-migratory, and radiating to the back. It was associated with two episodes of vomiting two days back without nausea and anorexia.

There was no hematemesis, abdominal distension, diarrhea, constipation, or bleeding per rectum. He did not have any chest pain, sweating, or pain radiating to his upper limbs. There was no dizziness, gait issues, weakness of limbs, seizures, or any other neurological symptoms. He denied any recent or chronic alcohol or drug ingestion, trauma or fall, or any history of changes in urine or stool color. There was no family history of sudden death, intracranial bleed, or aneurysm.

On examination, his blood pressure was 164/94 mm of Hg, heart rate was 84 beats per minute, and respiratory rate was 18 breaths per minute with 100% oxygen saturation on room air. The abdomen was symmetric on inspection and moving equally with respiration. There was no tenderness or rebound tenderness in any quadrants. Hernial orifices were intact, and genitalia were normal. Digital rectal examination revealed normal consistency fecal matter in the rectum with no malena, blood, or a palpable mass. Peripheral pulses were present and equal in both limbs and carotids.

Investigations

His electrocardiogram (ECG) and cardiac enzymes were normal. Chest X-ray (CXR) was normal. His white blood cell counts were 12,800 per microliter with 64% neutrophils. Electrolytes, renal function tests, liver function tests, and pancreatic enzymes were normal. Arterial blood gas analysis showed normal lactic acid level and acid-base balance.

**Figure 1:**
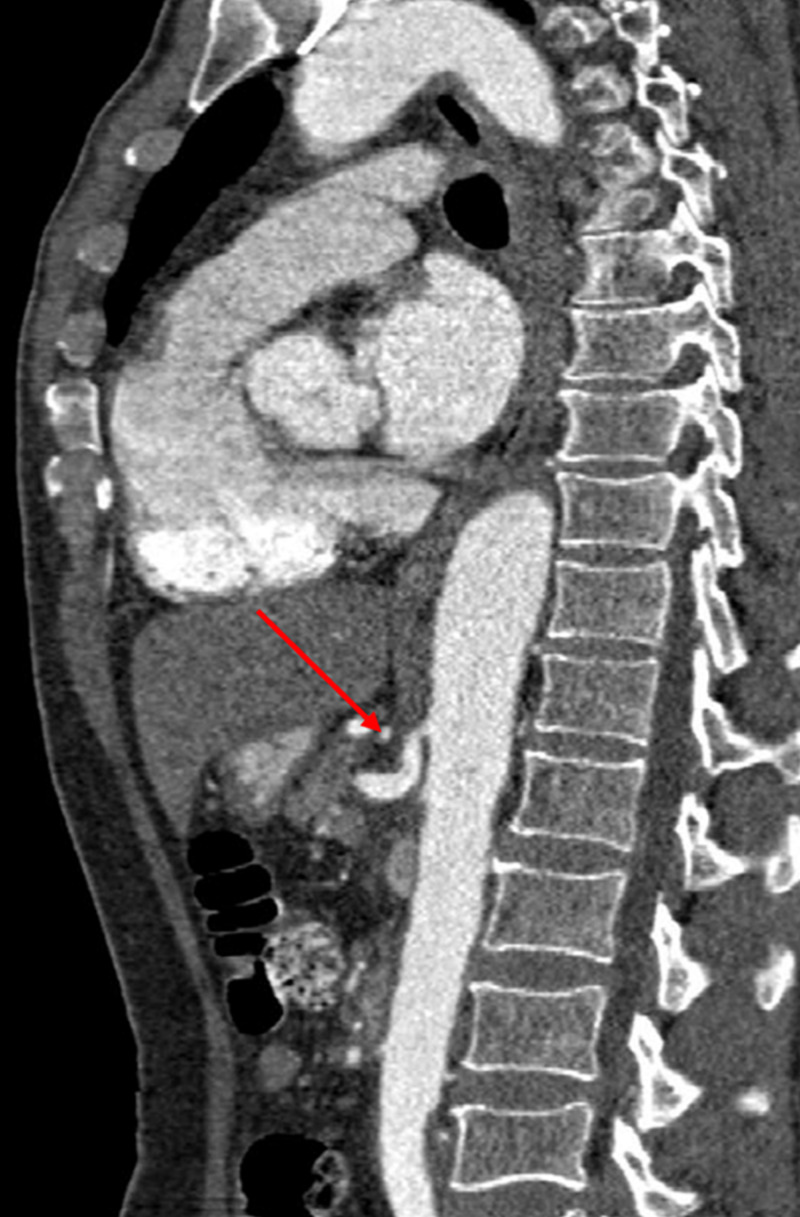
Sagittal reformatted CT aortogram image at the level of CA origin. Note the dissection in the proximal segment of CA (red arrow). CA, celiac artery

**Figure 2:**
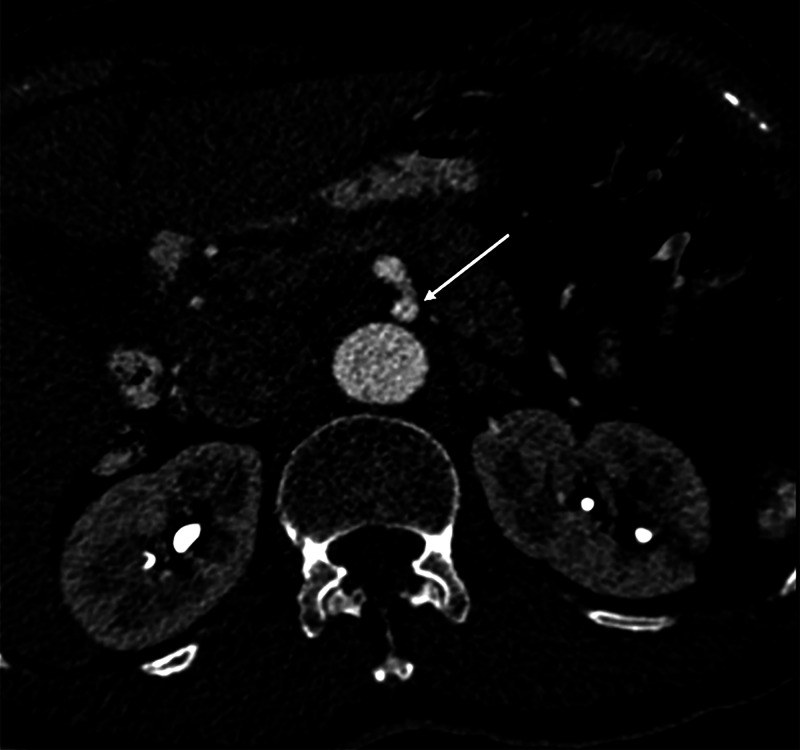
CT aortogram (axial image) demonstrating dissection of CA (white arrow) with patent true and false lumens. CA, celiac artery

**Figure 3:**
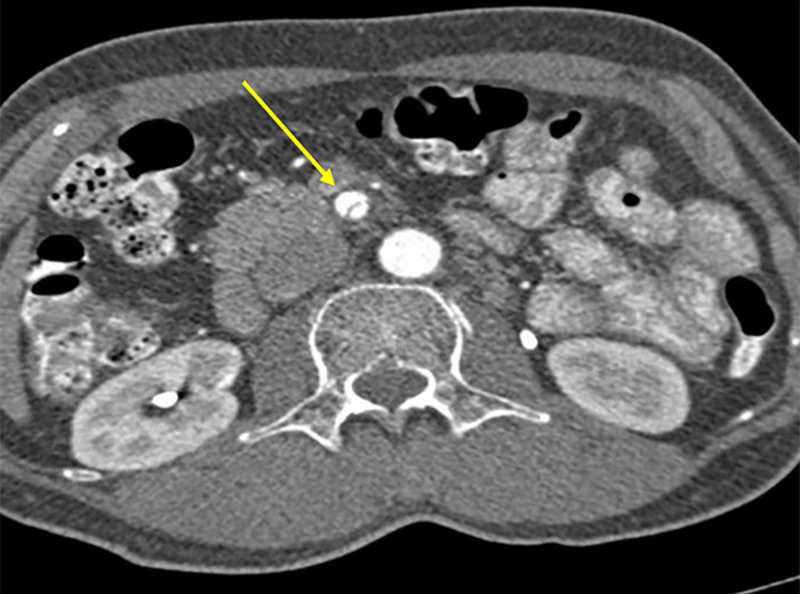
CT aortogram (axial image) demonstrating dissection of the SMA. SMA, superior mesenteric artery

**Figure 4:**
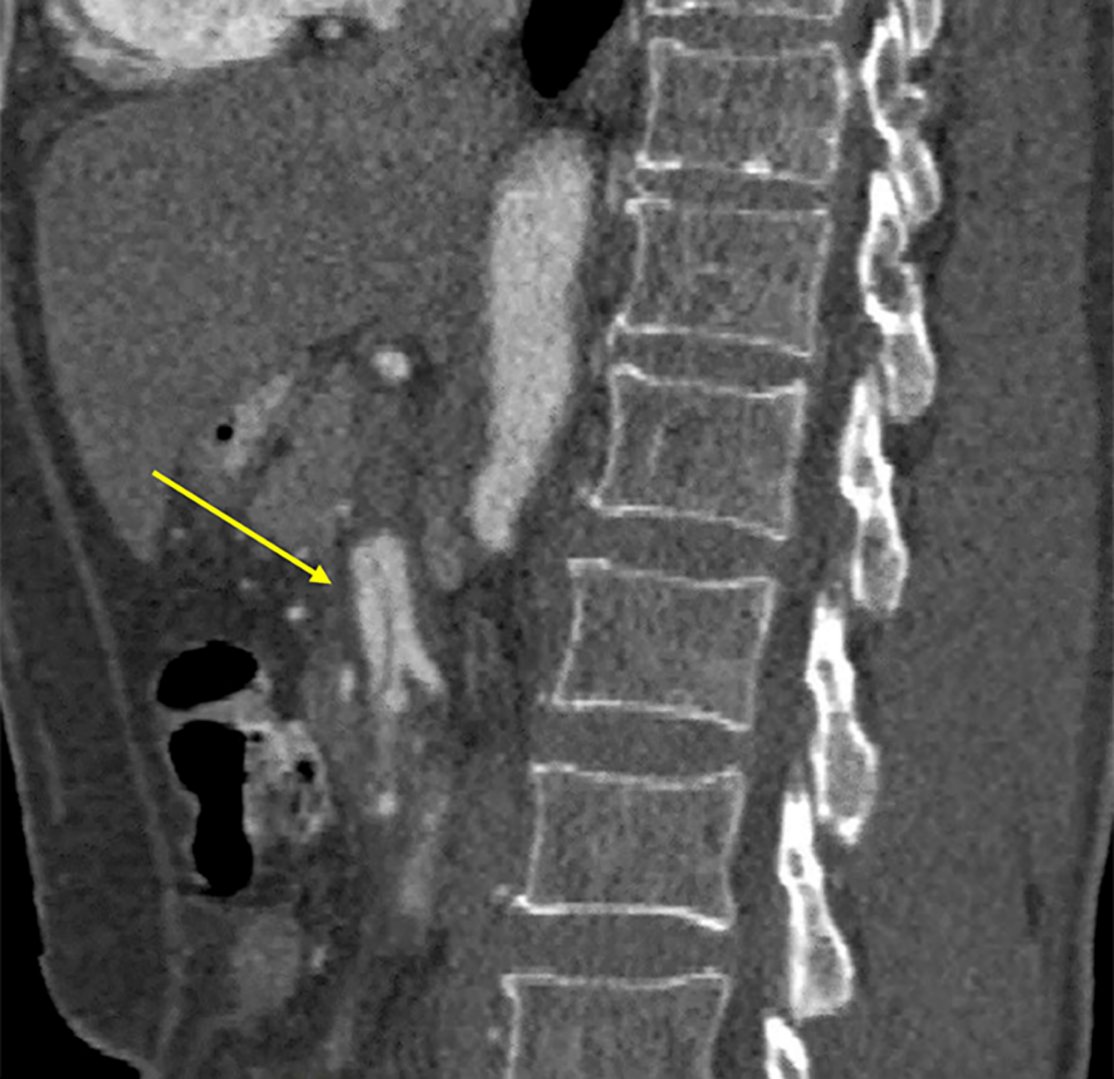
Sagittal reformatted CT aortogram image demonstrating dissection of the SMA. SMA, superior mesenteric artery

**Figure 5:**
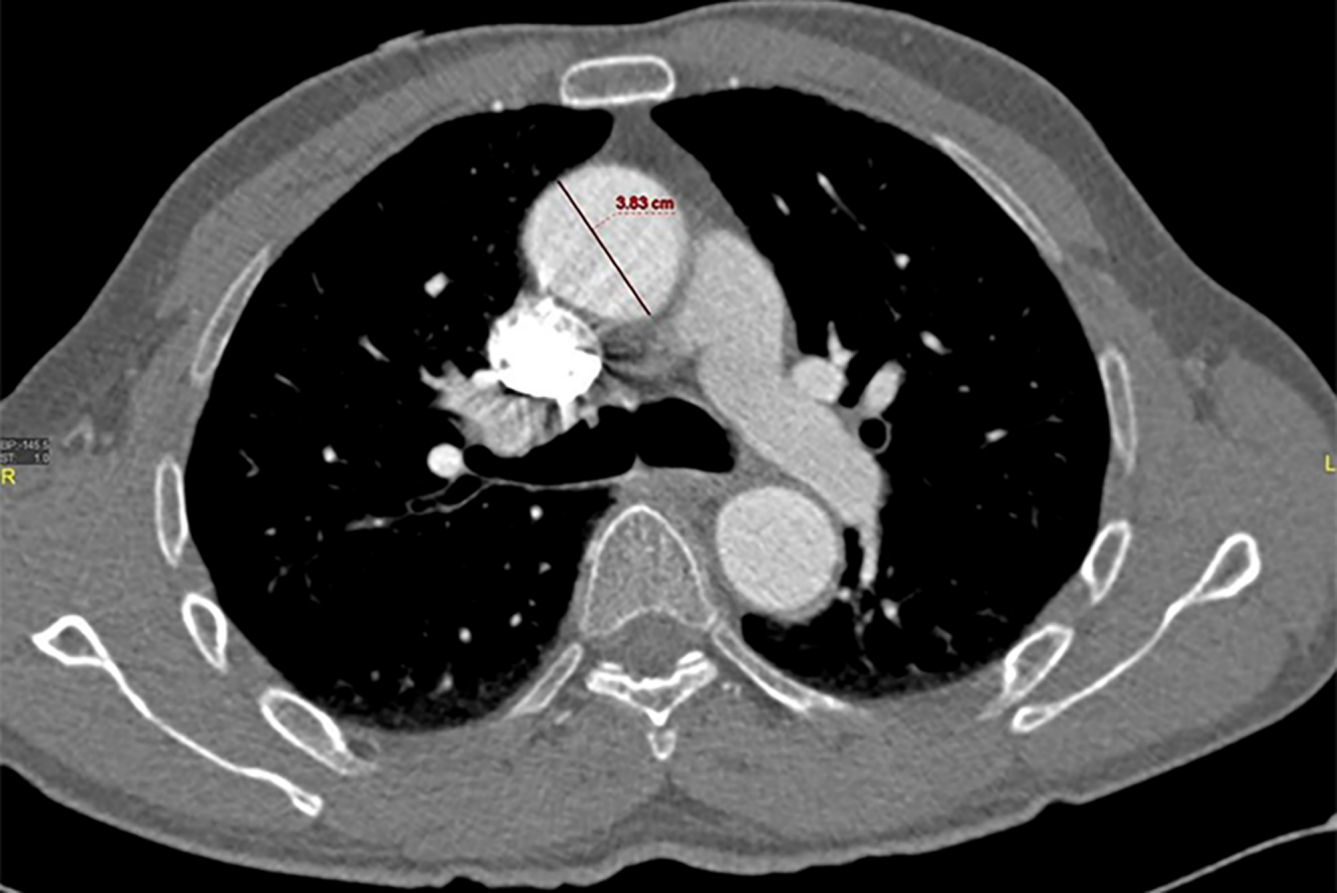
CT aortogram (axial image) demonstrating ectatic ascending aorta.

**Figure 6:**
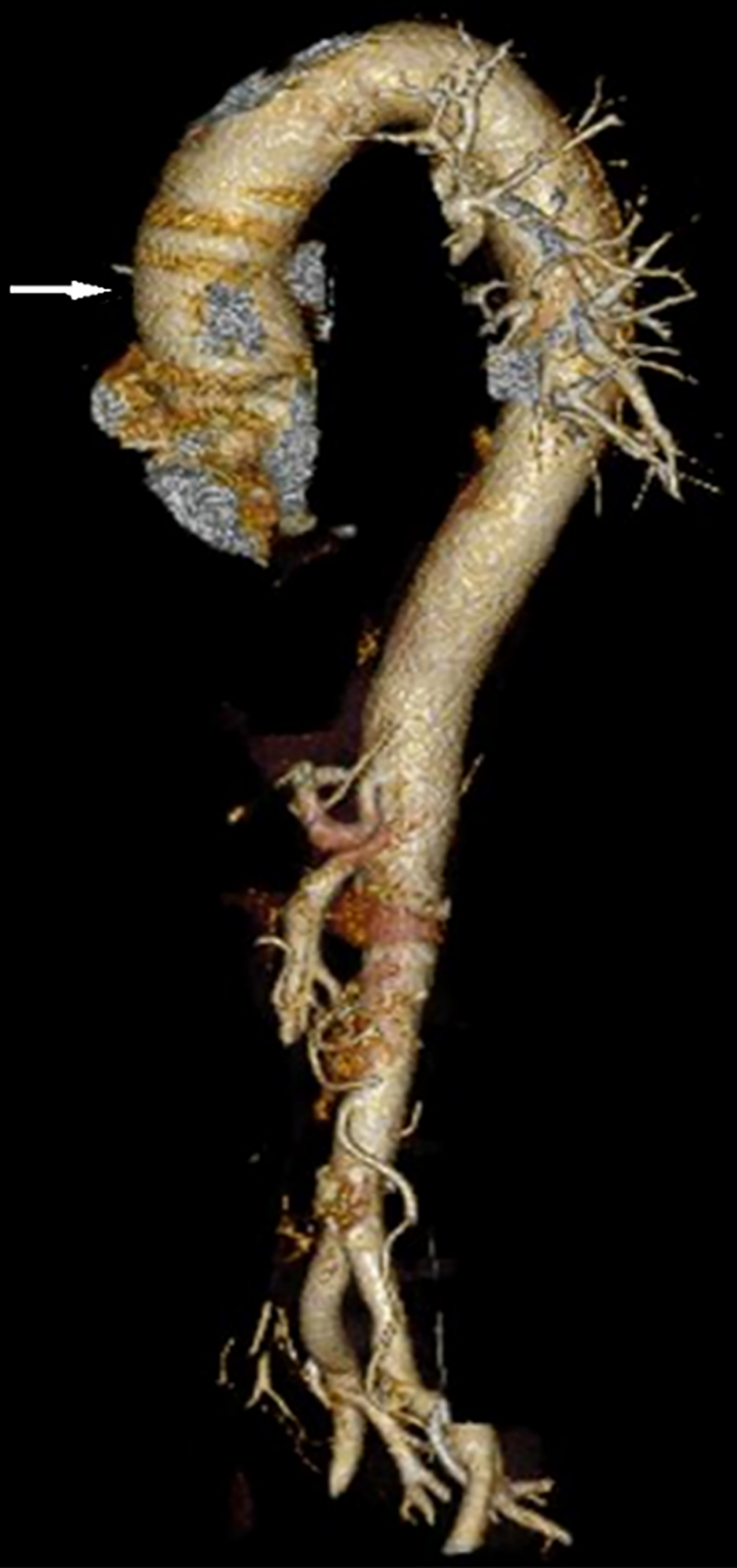
3D reconstruction of CT aortogram showing ectatic ascending aorta (white arrow).

CT angiography (CTA) revealed isolated dissection of SMA and CA, without ischemia. The dissection of CA was in the proximal part without involving its branches, and there was complete opacification of both true and false lumens (Figures 1, 2). The diameter of the true and false lumens of CA dissection was 1.8 mm and 2.3 mm, respectively. The dissection of the SMA was of approximately 3.3 cm in length, without involving its branches (Figures 3, 4). The diameter of the true and false lumens of SMA dissection was 4.1 mm and 4.9 mm, respectively. At the origin, the diameter of CA and SMA was 6 mm and 6.9 mm, respectively. Both dissections had patent false lumen with entry and re-entry representing type 1 dissection according to SMA dissection classification by Sakamoto et al. (Table 1) [4]. The ascending aorta was found to be ectatic with a diameter of 3.83 cm (Figures 5, 6). The infrarenal aortic diameter was 1.7 cm.

**Table 1:** Four types of SMA dissection classified by Sakamoto et al. [4]. ULP, ulcer-like projection; SMA, superior mesenteric artery

Type	Description
I	Patent false lumen with both entry and re-entry
II	“Cul-de-sac” shaped false lumen without re-entry
III	Thrombosed false lumen with ULP, which is defined as a localized blood-filled pouch protruding from the true lumen into the thrombosed false lumen
IV	Completely thrombosed false lumen without ULP

Differential diagnosis

Common causes of sudden onset epigastric pain are cardiac events, pneumothorax, perforated viscus, acute pancreatitis, acute hepatitis, gastritis, mesenteric ischemia, and aortic dissection.

An ECG, cardiac enzymes, and CXR helped to rule out acute cardiac and pulmonary conditions that require urgent treatment. A non-rigid abdomen with no tenderness and a CXR not showing any air under the diaphragm lowered the possibility of a perforated viscus. A patient presenting with severe, sudden onset epigastric pain should alert a clinician about acute pancreatitis, but a normal pancreatic amylase and lipase ruled it out as an active differential in our case. Normal liver enzymes, alkaline phosphatase, and serum bilirubin levels made acute hepatitis unlikely. Mesenteric ischemia and AD warranted a CT scan with IV contrast in stable patients and was promptly performed in our patient.

Treatment

The patient was treated with analgesia with non-steroidal anti-inflammatory drugs and opioids (as needed). Aspirin, clopidogrel, enoxaparin, and warfarin were started. Enoxaparin was continued for three days until the therapeutic level of warfarin was attained based on serial prothrombin time and international normalized ratio values. After three days of anti-coagulation and analgesics, his pain was relieved completely and was discharged home. His length of hospital stay was four days. During discharge, we prescribed warfarin and dual antiplatelet medications.

Outcome and follow-up

On his follow-up visit to the outpatient clinic, he was found to have complete resolution of symptoms without any recurrence. The patient received anticoagulation for six months after which a follow-up CT scan was performed, which revealed the persistence of ADs with no effect on vessel perfusion. There were no interval changes as compared to the previous CT scan. We discontinued anticoagulation after a total of six months of therapy. Another CT scan performed a year after initial presentation also showed no interval changes, denoting non-progression of the dissection. The patient was followed for another year with biannual visits to the outpatient department and did not develop any symptoms until the date of writing this report.

## Discussion

Isolated multiple visceral artery dissection (IMVAD) is a rare vascular disease mainly affecting men. It was first described in SMA by Bauersfeld in 1947 and in the CA by Foord and Lewis in 1959 [1,2]. Isolated extra-aortic AD mostly occurs in the renal and carotid arteries, but spontaneous IMVAD happens very rarely. Most of these dissections involve the SMA and CA [3].

Etiology

Its exact etiology is not clear. A review of the literature identifies risk factors such as hypertension, atherosclerosis, history of blunt or iatrogenic trauma, medial cystic degeneration, pregnancy, fibromuscular disease, and connective tissue disorders. However, some patients have no risk factors [5-8]. Another hypothesis to explain this phenomenon is “shear stress injury”. It is assumed that abnormal shear stress can develop at the transitional zone of SMA from a fixed (under the pancreas body) to a relatively unfixed (in the mesenteric root) segment. This abnormal shear stress can cause SMA dissection [6]. In our case, IMVAD was associated with ascending aortic ectasia. Whether they share a common etiology is unclear.

Presentation and diagnosis

It may present as an incidental finding on CT scan or with abdominal pain, which is the most common symptom reported. Other symptoms include nausea, vomiting, melena, and abdominal distention [9].

IMVAD is diagnosed on a CT scan; however, other modalities such as Doppler ultrasound may have a role in diagnosis [9]. AD poses a risk of bowel ischemia, the progression of dissection, rupture, and bleeding [3,5,7]. Therefore, a high index of suspicion is needed for its diagnosis.

Classification

Sakamoto et al. classified SMA dissection for the first time in 2007. This classification was based on the appearance of a false lumen (Table 1) [4].

There are also other classifications proposed for isolated SMA dissection. The classification provided by Zerbib et al. in 2010 divided SMA dissection into six types according to the presence of false luminal flow and true lumen patency at the dissected segment [10]. A similar classification is also proposed for isolated CA dissection by Sun et al. in 2016, which may help in guiding the treatment plan [11].

Management

Regarding the management of IMVAD, there are multiple treatment modalities. There is no consensus on the optimal management.

Observation

Observation alone was described as early as in 1948 and can be considered for incidental asymptomatic patients who have a non-progressive disease with close follow-up [3]. Yasuhara et al. described the first successful expectant management in SMA dissection in 1998 [12,13]. Since then, various authors have published their successful results.

Conservative Management

Conservative treatment in the form of anticoagulation and antiplatelet therapy appears an attractive initial option in uncomplicated cases, which results in the resolution of symptoms in most of the patients [14]. It was first reported by Ambo et al. in 1994 [15]. Another benefit of anticoagulation therapy, when used long term in patients who do not have ischemia, is the complete remodeling of the dissection. However, this effect is reported only in a few patients [14]. In our patient, there were no features of ischemia; therefore, we treated conservatively by antiplatelets and anticoagulation.

Currently, there is controversy regarding the recommended duration of anticoagulation. We opted to continue anticoagulation for six months and then follow with a CTA. The CTA scan after six months showed non-progression of the previous AD without any interval changes. Therefore, we discontinued anticoagulation and followed in the clinic.

Endovascular Treatment

Endovascular procedures and surgical interventions are also described, but mostly these options are reserved for the symptomatic and progressive disease [16]. The aim of endovascular stenting (EVS) is to seal the false lumen and re-establish the flow. It was first described by Leung et al. in 2000 [17]. EVS has the benefit of being less invasive as compared to open surgery and has a high procedural success rate and less morbidity. Both bare and covered stents are used. Covered stents are better for preventing enlargement or rupture of aneurysms, but these stent grafts can cover the branches, which can be detrimental [11]. EVS is usually indicated if the patient is not responding to conservative treatment. It may also be considered for recurrent symptoms, visceral malperfusion, or aneurysm. Although it is increasingly used, the long-term follow-up data is lacking. If the patient has features of bowel ischemia or aneurysm rupture, open surgery is performed.

Surgery

Open surgical options include thrombectomy, endoaneurysmorrhaphy, intimectomy and patch angioplasty, ligation of dissecting pseudoaneurysm, resection, venous bypass graft, and arterial bypass graft [9,11,18].

As there is a risk of progression of AD, close clinical and radiological follow-up is recommended [3,8].

## Conclusions

IMVAD is a rare vascular disease, and it should be considered in the differential diagnoses while evaluating a patient with severe abdominal pain. High degree of suspicion is the key to diagnosing IMVAD. CT scan is the standard diagnostic modality that helps not only in diagnosis and management but also assesses the progression of the disease and follow-up. Initial treatment should begin with anticoagulation and continued until there is disappearance of the dissection. Endovascular and surgical interventions are reserved for patients with severe, progressive disease and unremitting symptoms or patients with bowel ischemia.
